# Does low-density lipoprotein cholesterol induce inflammation? If so, does it matter? Current insights and future perspectives for novel therapies

**DOI:** 10.1186/s12916-019-1433-3

**Published:** 2019-11-01

**Authors:** Ruurt A. Jukema, Tarek A. N. Ahmed, Jean-Claude Tardif

**Affiliations:** 1 Department of Medicine, VU University Medical Centre Amsterdam, Amsterdam, the Netherlands; 2 Department of Medicine, Montreal Heart Institute, Université de Montréal, Montreal, Canada; 3 Department of Cardiology, Assiut University, Assiut, Egypt

**Keywords:** Inflammation, Cholesterol, Inflammasome, Immunity, Epigenetics, Atherosclerosis

## Abstract

**Background:**

Dyslipidemia and inflammation are closely interrelated contributors in the pathogenesis of atherosclerosis. Disorders of lipid metabolism initiate an inflammatory and immune-mediated response in atherosclerosis, while low-density lipoprotein cholesterol (LDL-C) lowering has possible pleiotropic anti-inflammatory effects that extend beyond lipid lowering.

**Main text:**

Activation of the immune system/inflammasome destabilizes the plaque, which makes it vulnerable to rupture, resulting in major adverse cardiac events (MACE). The activated immune system potentially accelerates atherosclerosis, and atherosclerosis activates the immune system, creating a vicious circle. LDL-C enhances inflammation, which can be measured through multiple parameters like high-sensitivity C-reactive protein (hsCRP). However, multiple studies have shown that CRP is a marker of residual risk and not, itself, a causal factor. Recently, anti-inflammatory therapy has been shown to decelerate atherosclerosis, resulting in fewer MACE. Nevertheless, an important side effect of anti-inflammatory therapy is the potential for increased infection risk, stressing the importance of only targeting patients with high residual inflammatory risk. Multiple (auto-)inflammatory diseases are potentially related to/influenced by LDL-C through inflammasome activation.

**Conclusions:**

Research suggests that LDL-C induces inflammation; inflammation is of proven importance in atherosclerotic disease progression; anti-inflammatory therapies yield promise in lowering (cardiovascular) disease risk, especially in selected patients with high (remaining) inflammatory risk; and intriguing new anti-inflammatory developments, for example, in nucleotide-binding leucine-rich repeat-containing pyrine receptor inflammasome targeting, are currently underway, including novel pathway interventions such as immune cell targeting and epigenetic interference. Long-term safety should be carefully monitored for these new strategies and cost-effectiveness carefully evaluated.

## Background

Although Rudolf Virchow introduced the hypothesis that inflammation, as well as cholesterol, plays a causal role in atherogenesis in the late 1800s, this assumption has lain dormant for almost a century. Over time, cholesterol has been considered the primary promoter of atherogenesis, and has been the main focus of research and drug development. It is only in the last few decades that the inflammation hypothesis has been resurrected, starting with Russell Ross postulating the “response to injury” hypothesis [1]. Since then, mechanistic understanding of the contributions of the innate and adaptive immune system to the atherosclerosis process and other diseases has led to a better understanding of the cross-talk between dyslipidemia and inflammation as key drivers of disease [2]. More recently, this has opened new avenues for therapeutics targeting atherosclerotic disease burden reduction [3–6]. In this opinion article, we highlight the contribution of inflammation to atherosclerosis in view of recent experimental and clinical trials, and we describe new frontiers to be explored in anti-inflammatory therapy, such as B and T cell function modification and epigenetics.

## Cross-talk between dyslipidemia and immunity

**Fig. 1:**
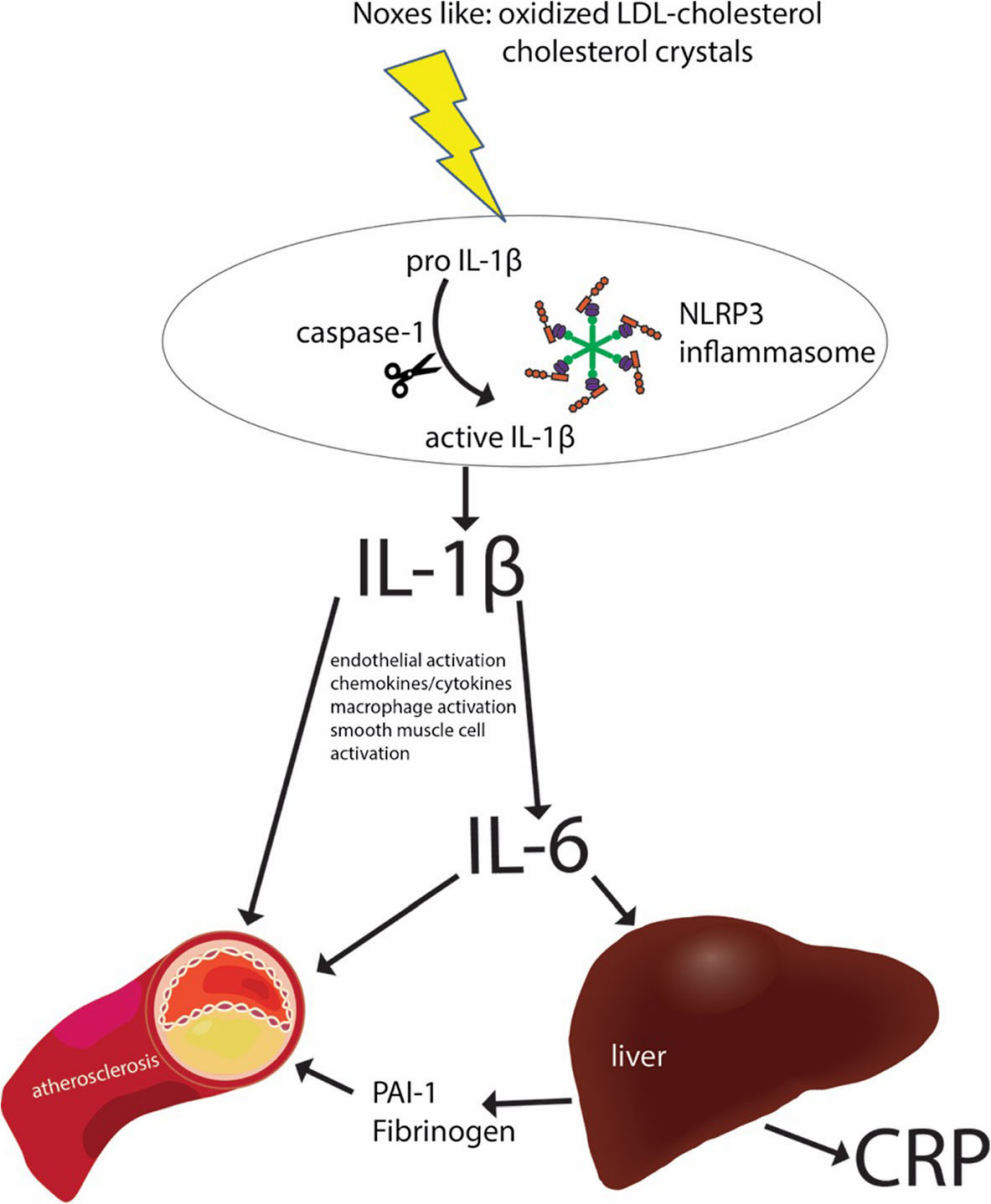
Inflammasomes are key signaling platforms that detect atherogenic microorganisms and sterile stressors, and that activate the highly pro-inflammatory cytokines interleukin (IL)-1β and IL-18. Inhibition of IL-1β/IL-6 signaling, which is initiated at the level of the nucleotide-binding leucine-rich repeat-containing pyrine receptor 3 (NLRP3) inflammasome, may therefore effectively reduce atherosclerotic CV outcomes. CRP C-reactive protein, LDL low-density lipoprotein, PAI-1 plasminogen activator inhibitor-1

Lipid metabolism has profound effects on both innate and adaptive immune systems through different mechanisms. Modified low-density lipoprotein cholesterol (LDL-C) enhances the activation of the innate immune system (i.e., first-line defense) by activating Toll-like receptor (TLR) pathways, which triggers pro-inflammatory signals [2, 6]. Through theses TLRs, cholesterol crystals, neutrophil extracellular traps, atheroprone flow, and hypoxia activate the nucleotide-binding leucine-rich repeat-containing pyrine receptor (NLRP3) inflammasome in the cytoplasm of macrophages located in the arterial intima (Fig. 1). The inflammasome is a protein complex that responds to noxious stimuli and cleaves pro-interleukin (IL)-1β and IL-18, which are then secreted as activated cytokines [2].

Conversely, inflammation modifies lipid metabolism. Both the innate and adaptive immune system regulate lipid metabolism, establishing a vicious cycle that promotes atherosclerosis. This is exerted through several mechanisms; for example, the cytokines released from immune system activation can influence fatty acid oxidation, and can activate lipoprotein lipase in adipose and muscle tissue, and hepatic lipase, leading to dyslipoproteinemia [4]. It should be noted that the inflammatory component of atherosclerosis is a chronic hyper-inflamed systemic response rather than local vascular dysfunction.

## Inflammatory biomarkers and atherosclerosis

The contribution of various biomarkers to predicting the risk of clinical atherosclerotic cardiovascular (CV) events has been studied not only in patients with CV disease, but also in apparently healthy individuals. The concept of “personalized medicine” or “targeted therapy” has been proposed, in which lipid-lowering and/or anti-inflammatory treatment plans are tailored based on basal levels of these inflammatory markers.

Among the diverse group of circulating biomarkers, C-reactive protein (CRP) stands out as a leading biomarker for risk prediction. CRP measured by high-sensitivity assay (hsCRP) can independently predict CV events. A primary prevention trial, the Justification for the Use of Statins in Prevention: an Intervention Trial Evaluating Rosuvastatin (JUPITER) trial, showed that rosuvastatin reduced the rate of first major cardiovascular events (MACE) by 47% in patients with LDL-C levels of < 130 mg/dL and hsCRP of > 2 mg/L [7]. However, Mendelian randomization and animal studies disproved a causal relationship between CRP and atherosclerotic events [3, 5]. This all indicates that CRP is a valuable biomarker for atherosclerotic risk/inflammation estimation, but that it is not a causal factor.

In contrast to CRP, there are consistent reports from multiple studies indicating a causal effect of IL-6 signaling in atherosclerosis. This has been related to plaque destabilization, microvascular dysfunction, and acute CV events (Fig. 1) [8, 9]. As of yet, IL-1β, the primary circulating form of IL-1 (the most powerful inducer of innate immunity), cannot reliably be measured in plasma. Accordingly, there are no comparable epidemiologic studies relating IL-1β to CV risk [10].

## Lipid-lowering treatment and anti-inflammation: is there a causal relationship?

The anti-inflammatory effects of lipid-lowering therapy have long been controversial. Whether the benefits, in terms of primary or secondary prevention of CV events, reflect a mere effect of LDL lowering or whether lipid-independent anti-inflammatory actions prevail has been a point of continuous debate. We still do not know how low lipoprotein levels need to go to attain a sufficient anti-inflammatory response, or whether the addition of specific anti-inflammatory therapy adds further risk reduction beyond lipid lowering.

### Statins and anti-inflammation

Experimental studies have illustrated that statins promote the expression of anti-inflammatory and cytoprotective molecules in the endothelium [11]. Statins also modulate the adaptive immune system by suppressing pro-inflammatory responses of T cells [12]. Human studies have provided further exploration of the anti-inflammatory effects of statins. An intriguing observation from the JUPITER study [13] was that rosuvastatin reduced venous thrombosis, even though there are no atherosclerotic plaques to rupture in the venous wall and LDL-C has little influence on stasis-induced thrombosis [14]. Further, the greatest statin-attributed absolute risk reductions in JUPITER were observed among those with the highest baseline levels of inflammation [15]. However, results from the Prospective Study of Pravastatin in the Elderly at Risk (PROSPER) trial suggested that CRP had only modest value in predicting CV risk or response to statin therapy among the elderly [16]. Thus, data from experimental and clinical studies support the anti-inflammatory and immunomodulatory effects of statins. Nevertheless, it cannot be demonstrated unequivocally whether this is a direct effect or merely due to LDL-C reduction.

### Non-statin lipid-lowering drugs and anti-inflammation

It has been observed that the anti-inflammatory effects of lipid lowering extend beyond statins to other lipid-lowering drugs, supporting the notion that the anti-inflammatory response is not an exclusive class effect, but rather a response to lipid reduction. Ezetimibe reduces plasma levels of inflammatory markers [17] and diminishes plaque inflammation in atherosclerosis animal models [18]. In the Improved Reduction of Outcomes: Vytorin Efficacy International Trial (IMPROVE-IT), combinations of statins and ezetimibe were associated with better outcomes, achieving dual targets relating to both CRP and LDL-C rather than meeting only LDL-C targets [19]. For the new lipid-lowering class of proprotein convertase subtilisin/kexin type 9 (PCSK9) inhibitors, the anti-inflammatory effects are less clear [3, 20, 21]. A role of PCSK9 in the development of atherosclerosis and vascular wall inflammation has been hypothesized [22–24]. Data from experimental studies have shown that PCSK9 in the atherosclerotic plaques regulates the expression of genes controlling inflammation through both LDL-dependent and LDL-independent pathways [25–28]. Interestingly, large CV outcome trials of PCSK9 inhibitors have demonstrated a lack of effect on CRP levels, emphasizing a possible anti-inflammatory effect of PCSK9 inhibitors independent of the CRP pathway [28].

## Inflammation as a novel therapeutic target for atherosclerotic CV disease: insights from recent clinical trials

The hypothesis that pharmacological modulation of inflammation can reduce CV events is controversial. Recently, the Canakinumab Anti-inflammatory Thrombosis Outcome Study (CANTOS) shed new light on this issue as a proof-of-principle study. Canakinumab is a high-affinity IL-1β monoclonal antibody that binds and functionally neutralizes the bioactivity of this pro-inflammatory cytokine. In CANTOS, patients were randomized to one of three doses of canakinumab or placebo [29]. All patients received standard of care therapy. Canakinumab administration resulted in MACE reduction. However, CV mortality was not reduced at any dose. Moreover, canakinumab was associated with a higher incidence of fatal infections compared to placebo. In a sub-analysis from the study, MACE were significantly reduced in patients establishing hsCRP levels of < 2 mg/L but not in those establishing hsCRP levels of > 2 mg/L after 3 months of treatment, indicating those who would probably benefit most from ongoing treatment after the first dose [30]. Thus, CANTOS represented the first major proof-of-concept trial for targeting inflammation in atherosclerosis. This has triggered attempts to seek alternative potential avenues for inflammation inhibition: the inflammatory signaling pathway is complicated and redundant, so blocking a single mediator (such as IL-1β) may not block all the inflammatory pathways implicated in atherogenesis.

Parallel to CANTOS, the Cardiovascular Inflammation Reduction Trial (CIRT) assessed low-dose methotrexate in the secondary prevention of atherothrombotic events among patients with a history of myocardial infarction or multivessel coronary artery disease who additionally had either type-2 diabetes or metabolic syndrome. This trial showed that low-dose methotrexate did not reduce levels of IL-1β, IL-6, or hsCRP and was not associated with fewer MACE than placebo among patients with atherosclerosis whose condition was stable but who were at high risk of CV events.

The contradicting results from these two contemporary trials, CANTOS and CIRT, raise a potential hypothesis that adopting anti-inflammatory treatment in the prevention of atherosclerotic CV disease largely depends on the mediators targeted along the rather complex inflammatory cascade. Inflammasomes are key signaling platforms that detect atherogenic microorganisms and sterile stressors, and activate the highly pro-inflammatory cytokines IL-1β and IL-18. Inhibition of IL-1β and IL-6 signaling, which is initiated at the level of the NLRP3 inflammasome [31], did effectively reduce atherosclerotic CV outcomes in CANTOS (Fig. 1). While CANTOS targeted patients with residual inflammatory risk (median baseline hsCRP 4.2 mg/L), CIRT did not screen for CRP level among participants (median baseline hsCRP 1.6 mg/L).

Finally, the Low-Dose Colchicine (LoDoCo) trial tested colchicine in a small randomized trial of patients with stable CAD [32]; primary results are yet to be confirmed in the larger LoDoCo2 trial (ACTRN12614000093684) and Colchicine Cardiovascular Outcomes Trial (COLCOT; NCT02551094).

## (Cholesterol-induced) inflammation, diseases other than atherosclerosis, and effects of cholesterol lowering by statins

As argued, inflammation/inflammasome activation and atherosclerosis are closely and probably causally related, but is there also evidence for a relationship between (cholesterol-induced) inflammation/inflammasome activation and other diseases with inflammatory aspects? This may be the case. Statins have been described to have cholesterol-lowering as well as anti-inflammatory properties and may be advantageous in such conditions, making this class of drugs attractive in multiple diseases. However, this double-edged action is also making it difficult to evaluate if the observed beneficial properties result from cholesterol-lowering properties only, from immunomodulation properties only, or from both with possible interactions between the two [33].

On the one hand, classic atherosclerosis may thus be in part the result of “low-grade inflammation,” marked by mildly elevated CRP levels. On the other hand, systemic “high-grade inflammation” underlying autoimmune diseases, for example, rheumatic diseases or systemic lupus erythematosus, are associated with accelerated atherosclerosis and various types of vasculopathies [34, 35]. Because inflammation is the common and driving risk factor in this respect, the main focus should be to control inflammation through low-dose corticosteroids, biologics, or immunosuppressive agents. However, cholesterol-lowering therapy has also been reported to be beneficial in these conditions [36].

Multiple auto-inflammatory diseases have been described in which the inflammasome plays an important role [37]. Familial Mediterranean fever and cryopyrin-associated periodic syndromes are both examples of auto-inflammatory diseases characterized by increased activity of the inflammasome leading to overproduction of IL-1β, which is responsible for the clinical manifestations of these diseases. The development of anti-IL-1β therapy led to successful treatments for both syndromes [38]. However, no clear role for cholesterol as a driver or cholesterol-lowering therapy as a treatment has been described yet for these diseases.

Diabetes mellitus (DM) is unequivocally associated with accelerated atherosclerosis. Patients with type 2 DM have elevated NLRP3 levels in monocytes, increased activity of the inflammasome, and high blood levels of IL-1β and IL-18. Studies have shown that the progression of atherosclerosis in DM involves inappropriate, persistent inflammation induced through excessive inflammasome activation by pathogens and endogenous danger stimuli [39]. Two months of metformin therapy significantly inhibited the maturation of IL-1β in monocyte-derived macrophages from patients with type 2 DM [40]. Beyond its hypoglycemic effect, the anti-inflammatory effect of metformin might inhibit the progression of atherosclerosis [40]. Cholesterol lowering with statins, simultaneously reducing CRP, has proven particularly effective in reducing clinical atherosclerotic events in patients with DM [41].

Since the introduction of effective antiretroviral therapy in the treatment of human immunodeficiency virus (HIV), focus has shifted to preventing HIV-associated comorbidities. Atherosclerosis-associated CV disease is one of the leading causes of mortality among HIV patients [42]. There is growing evidence indicating that HIV infection, whether productive or latent, and subsequent inflammatory processes accelerate atherosclerosis [43]. Antiretroviral therapy reduces atherothrombotic events in HIV patients, but it is not enough to sufficiently prevent CV disease [44].

As shown, LDL-C induces inflammasome activation, leading to the production of IL-1β and IL-18, which accelerate atherosclerosis. Multiple diseases have been linked to enhanced IL-1β and IL-18 and/or inflammasome activation as discussed above. Therefore, the very recent observation that a genetic variation in the NLRP3 inflammasome gene is associated with not only CV but also total mortality is intriguing (hotline presentation by W. März at the European Atherosclerosis Society, Maastricht, May 2019). One could argue that the course of these inflammatory diseases could indeed be influenced by or associated with LDL-C levels, considering the partly similar pathogenesis. However, although links with enhanced atherosclerosis have been described, no solid evidence is yet found in the literature for a direct causal relationship between LDL-C, inflammasome activation, and the course of these diseases themselves. Clearly, more research is required.

## Residual inflammatory risk versus residual cholesterol risk: no more coin flipping

High-dose statin therapy and other contemporary standard therapies, including potent novel anti-platelets, can only prevent a fraction of events associated with residual burden; These rather refractory/recalcitrant events represent an intriguing unmet medical challenge. This shifts treatment decisions from the flip of a coin to the era of “precision medicine” [45], in which treatment plans are tailored based on readily measured biomarkers. Those with LDL-C levels above the target levels despite the maximum-tolerated statin therapy are considered to have residual cholesterol risk, and should have either ezetimibe and/or a PCSK9 inhibitor added to their treatment [46]. Those who have persistently high inflammation burden, as demonstrated by a persistent hsCRP > 2 mg/L, are considered to have residual inflammatory risk, and could be assigned to an evidence-based anti-inflammatory treatment such as canakinumab if demonstrated safe over the long term. Both strategies lead to an additional relative risk reduction of 15%.

**Fig. 2:**
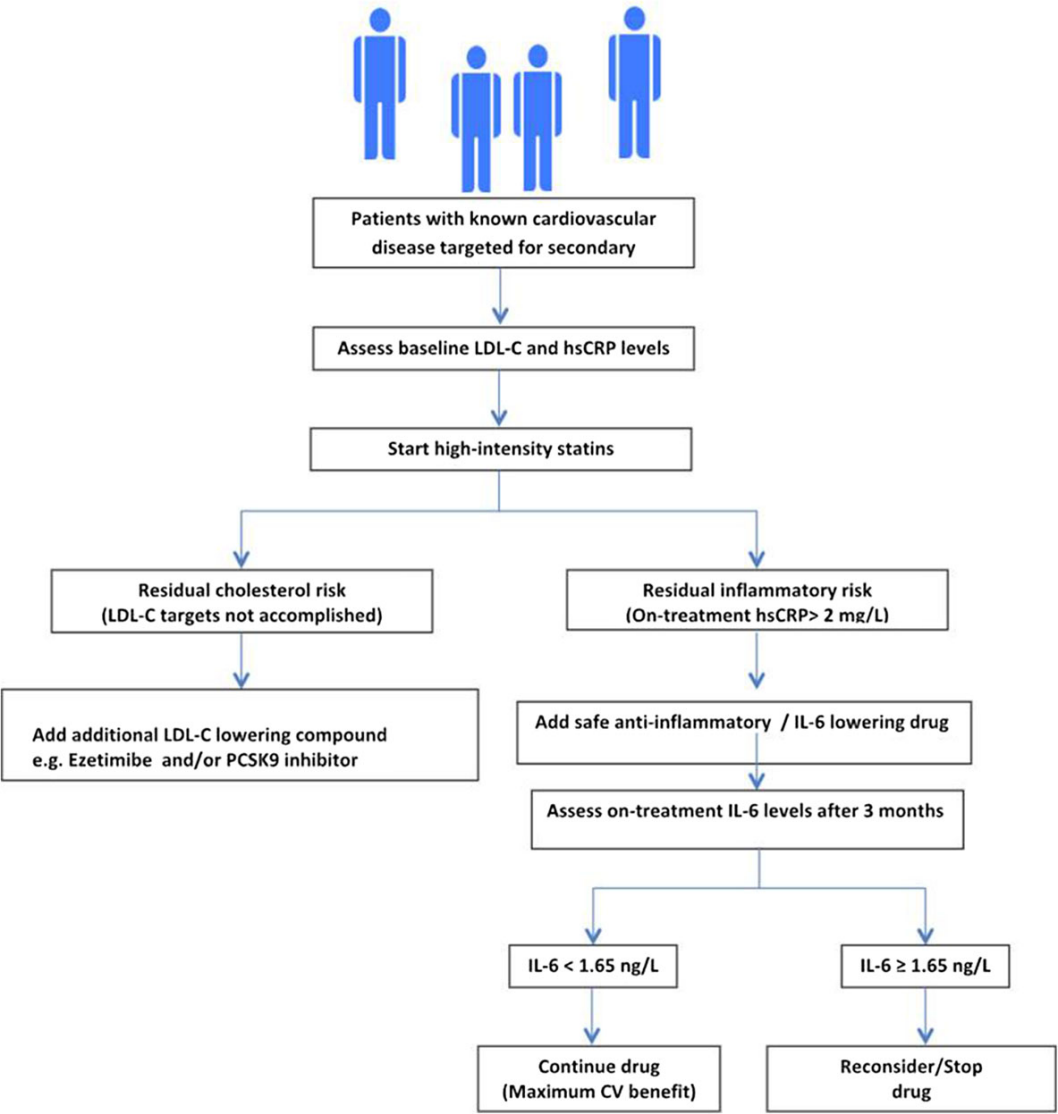
Tentative algorithm for guiding system of care selection of the appropriate treatment customized to the patient’s residual cholesterol versus inflammatory risk, when long-term anti-inflammatory therapy appears to be safe. CV cardiovascular, hsCRP high-sensitivity C-reactive protein, IL interleukin, LDL-C low-density lipoprotein cholesterol, PCSK9 proprotein convertase subtilisin/kexin type 9

A recent pre-specified sub-study of CANTOS addressed the degree of IL-6 reduction and its relation to CV event reduction [47]. Over a follow-up period extending to 5 years, patients on canakinumab who achieved on-treatment IL-6 levels below the study median value (1.65 ng/L) experienced a 32% reduction in MACE and a 48% reduction in all-cause mortality. Those with on-treatment IL-6 levels ≥1.65 ng/L had no significant benefit for these end-points. To optimize patient care, based on previous data and if long-term anti-inflammatory therapy appears safe, we propose an algorithm (Fig. 2) for guiding system of care selection of the appropriate treatment, customized to the patient’s residual risk. There is still a lack of evidence regarding the effect of combining non-statin lipid-lowering medications and novel anti-inflammatory therapy among patients with combined residual risk. This remains to be elucidated in future clinical studies. Cost-effectiveness will become an important issue here [48, 49].

## Discussion

Referring back to the main question addressed by our opinion article: does LDL-C induce inflammation? The answer is yes, it does. Now, does this matter? Again, yes it does.

It is now evident that LDL-C lowering, especially with statins, reduces inflammation and retards atherosclerosis progression. New therapeutic challenges still lie ahead, that will be aided by revolutionary concepts regarding the fundamentals of the biological features of atherosclerosis and the revisited role of inflammation. Preclinical studies and Mendelian randomization studies paved the way toward establishing a causal link between inflammation and atherosclerosis. The CANTOS represented the strongest proof-of-principle clinical study to support the inflammatory hypothesis of atherosclerosis. However, the latest data from the CIRT, with its negative results, imply that the inflammatory pathway is rather complex and that the adenosine-mediated anti-inflammatory effect of methotrexate might not be as effective as canakinumab at targeting IL-1β. IL-1β represents the tip of the iceberg; there are several other potential inflammatory mediators that could be directly targeted to halt atherosclerosis progression, such as by directly targeting the inflammasome, IL-1α, IL-6, IL-18, and IL-33, among others. The redundant inflammatory signaling pathway raises the possibility of a complementary, rather than supplementary, role for anti-inflammatory treatments, anticipating a sort of “sequential/cumulative multi-level mediator blocking” along the inflammatory pathway. Diseases other than atherosclerosis with an inflammatory component may be targets for a combined lipid/inflammation-lowering strategy. The results of CANTOS encourage further clinical studies evaluating other inflammatory mediators in different clinical settings of secondary prevention, but hurdles still have to be overcome and new avenues explored.

### Are we targeting the right patient and the right (stage of) disease: future directions

It is noteworthy that patients in the acute state of myocardial infarction, who have marked inflammation and plaque instability, were excluded from CANTOS and CIRT, which included patients at least 30 days from the index event [49, 50]. IL-6 contributes to atherosclerotic plaque destabilization and is thought to be involved in myocardial injury during ischemia– reperfusion. In a study assessing the circulating levels of IL-6 receptors and their relation to long-term clinical outcomes in patients with ST-segment elevation myocardial infarction, patients in the highest quartile of IL-6 levels had decreased event-free survival and higher mortality compared with the other lower three quartiles [51].

In a randomized trial among patients with non-ST-segment elevation myocardial infarction, a single intravenous dose of tocilizumab, an IL-6 antagonizing antibody, administered prior to percutaneous coronary intervention attenuated the inflammatory response by 50%, and reduced the myocardial injury as evidenced by the percutaneous coronary intervention-related troponin T release [52]. An ongoing clinical trial by the same investigators is assessing the effect of anti-IL-6R treatment with tocilizumab in the setting of a ST-segment elevation myocardial infarction (ASSAIL-MI trial; NCT03004703).

Experimental studies have shown that downregulation or inhibition of one or more of the inflammasome components through small interfering RNA reduces the infarct size and preserves myocardial contractility [53, 54]. Toldo and Abbate [55] have reached the conclusion that the window of opportunity for intervening using an NLRP3 inflammasome inhibitor is between 1 and 3 h from the onset of reperfusion, suggesting that the expression and activation of NLRP3 inflammasome is at its highest in this timeframe. Data on the role of the NLRP3 inflammasome in patients with an acute myocardial infarction or other inflammatory diseases are still scarce. Several experimental drugs targeting different steps along the inflammasome activation cascade are being investigated, including colchicine.

**Fig. 3:**
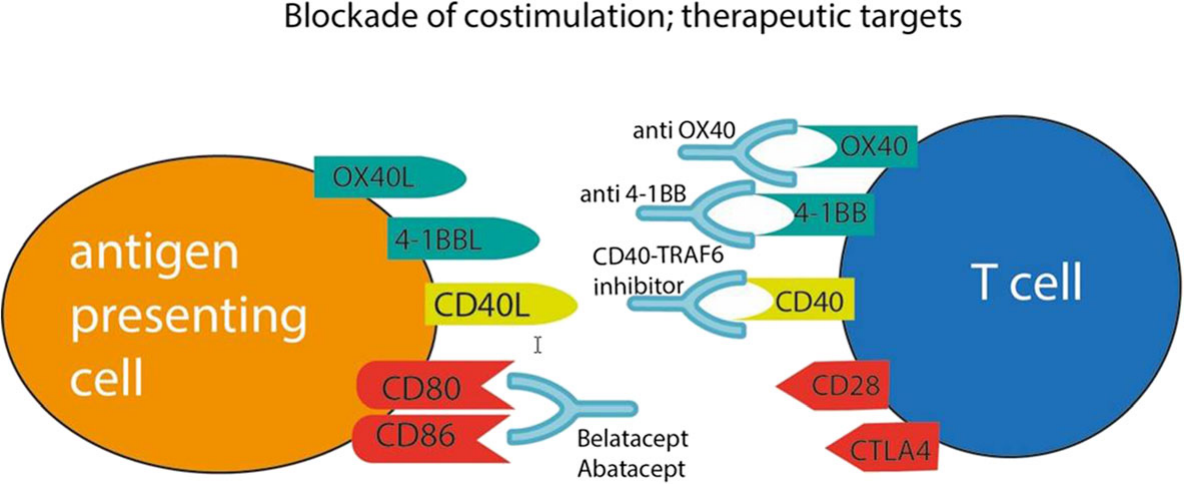
Co-stimulation blocking as a therapeutic target to diminish atherosclerosis. Antigen presenting cell (orange) and T cell (blue) are depicted with (a selection of) their receptors. Therapeutic antibodies that may block these receptors are shown in light blue

In the search for novel anti-inflammatory strategies, immune cells have been identified as pivotal elements contributing to plaque development and dynamics [56]. Interest is growing in innate immune cells in light of the recent notion that innate immunity, for a long time considered to be incapable of eliciting an adaptive response (i.e., could not be trained), does actually exhibit immunological memory mediated via epigenetic reprogramming. Risk factors for atherosclerosis promote immune cell migration by pre-activating circulating innate immune cells. Subsequently, innate immune cell activation, mediated by metabolic and epigenetic reprogramming, may perpetuate a systemic low-grade state of inflammation in atherosclerosis. This knowledge gives rise to new therapeutic modalities in which epigenetic or metabolic modulation of innate immune cells may lead to a decreased rate of chronic systemic inflammation, thereby alleviating atherosclerosis as well as its associated morbidities [56]. A recently proposed anti-inflammatory mechanism is the therapeutic targeting of co-stimulation [57, 58]. By blocking this co-stimulation of antigen presenting cells or T cells, atherosclerosis could be mitigated (Fig. 3) [58, 59]. The effects of these co-stimulation blockers on atherosclerosis should be investigated in future clinical studies. Anti-CD80/86 treatment with cytotoxic T lymphocyte antigen–immunoglobulin, such as the US Food and Drug Administration-approved abatacept and belatacept, and cluster of differentiation 40–tumor necrosis factor receptor associated factor 6 inhibitors have already been developed, but blocking of tumor necrosis factor receptor superfamily, member 4(OX40)as well as anti tumor necrosis factor receptor superfamily member 9 (4-1BB) co-stimulation could be interesting targets. However, it should be taken into consideration that blocking co-stimulation could lead to serious adverse effects. In the context of the complex interplay of the immune system in atherosclerosis, B cells also represent an attractive therapeutic target against atherosclerosis. They exert strong protective and detrimental effects on atherosclerosis progression. Establishing protective B cell antibody responses is a tempting strategy in halting atherosclerosis progression, either by active vaccination or by passive immunoglobulin transfer [60]. Recent research has increasingly recognized epigenetic mechanisms associated with atherosclerotic disease. These mechanisms include DNA methylation/demethylation, histone acetylation/deacetylation, and non-coding RNAs [61]. Analysis of human atherosclerotic plaques has demonstrated global DNA hypermethylation, which suggests a strong link between DNA methylation and atherosclerosis development [62]. A recent study analyzing whole-genome epigenetic factors implicated in atherogenesis identified tissue-specific enhancer chromatin regions that probably regulate the transcription of angiopoietin/angiopoietin-like genes that play a crucial role in atherosclerosis and angiogenesis [63]. This sparked the development of new epigenetic drugs targeting chromatin architecture to halt atherosclerosis. Among these, histone deacetylase inhibitors have demonstrated some preclinical efficacy in experimental models [64, 65]. Technical advances in biological approaches such as RNA-sequencing and DNA profiling have paved the way toward an anticipated leap in the development and validation of epigenetic drugs over the coming decades.

Finally, what remains of concern is the safety profile of the emerging anti-inflammatory and immune-modulating medications. The higher rate of fatal infections with canakinumab and the pro-atherogenic properties of IL-6 inhibitors should be carefully considered. Long-term post-marketing surveillance studies should be continuously conducted to note any potential hazards. Long-term experience with the application of these anti-inflammatory medications in rheumatologic diseases might provide us with a road map as we approach widespread clinical application in the CV field.

## Conclusions

Based on the presented evidence we conclude that LDL-C induces inflammation; inflammation is of proven importance in atherosclerotic disease progression; anti-inflammatory therapies show promise in lowering (CV) disease risk, especially in selected patients with high (remaining) inflammatory risk; and intriguing new anti-inflammatory developments, for example, in NLRP3 inflammasome targeting, are currently underway, including novel pathway interventions such as immune cell targeting and epigenetic interference. Long-term safety should be carefully monitored for these new strategies and cost-effectiveness carefully evaluated.

## Data Availability

All data is reported in the manuscript.

## Abbreviations

CRP: C-reactive protein
CV: Cardiovascular
DM: Diabetes mellitus
hsCRP: High-sensitivity C-reactive protein
IL: interleukin
LDL-C: Low-density lipoprotein cholesterol
MACE: Major adverse cardiac event
NLRP3: Nucleotide-binding leucine-rich repeat-containing pyrine receptor 3
PCSK9: Proprotein convertase subtilisin/kexin type 9
TLR: Toll-like receptor
